# Enhanced Fermentative Hydrogen and Methane Production from an Inhibitory Fruit-Flavored Medium with Membrane-Encapsulated Cells

**DOI:** 10.3390/membranes5040616

**Published:** 2015-10-16

**Authors:** Julius Akinbomi, Rachman Wikandari, Mohammad J. Taherzadeh

**Affiliations:** Swedish Centre for Resource Recovery, University of Borås, 501 90 Borås, Sweden; E-Mails: Rachman.Wikandari@hb.se (R.W.): mohammad.taherzadeh@hb.se (M.J.T.)

**Keywords:** encapsulated bacteria, fruit flavors, membrane, hydrogen, methane, inhibition

## Abstract

This study focused on the possibility of improving fermentative hydrogen and methane production from an inhibitory fruit-flavored medium using polyvinylidene fluoride (PVDF) membrane-encapsulated cells. Hexanal, myrcene, and octanol, which are naturally produced in fruits such as apple, grape, mango, orange, strawberry, and plum, were investigated. Batch and semi-continuous fermentation processes at 55 °C were carried out. Presence of 5 g/L of myrcene, octanol, and hexanal resulted in no methane formation by fermenting bacteria, while encapsulated cells in the membranes resulted in successful fermentation with 182, 111, and 150 mL/g COD of methane, respectively. The flavor inhibitions were not serious on hydrogen-producing bacteria. With free cells in the presence of 5 g/L (final concentration) of hexanal-, myrcene-, and octanol-flavored media, average daily yields of 68, 133, and 88 mL/g COD of hydrogen, respectively, were obtained. However, cell encapsulation further improved these hydrogen yields to 189, 179, and 198 mL/g COD. The results from this study indicate that the yields of fermentative hydrogen and methane productions from an inhibitory medium could be improved using encapsulated cells.

## 1. Introduction

Increasing energy demand and the depletion of fossil fuel reserves, coupled with global warming, have stimulated a rapid growth in developing alternative energy sources that are sustainable, renewable, and environmentally friendly. Energy carriers such as hydrogen and methane have been suggested as good substitutes for fossil fuels. Accordingly, various production pathways have been explored for hydrogen and methane productions including water electrolysis (power-to-gas), thermo-chemical processing, photo-chemical processing, photo-catalytic processing, and photo-electrochemical processing [1], as well as biological methods including photo-fermentation [2,3] and anaerobic fermentation [4,5]. Among the diverse production pathways, anaerobic fermentation via dark fermentation for hydrogen and methane production seems to be a promising option because of its low energy requirement and renewable and non-polluting qualities, as well as its ability to utilize organic residuals as carbon and energy sources. However, the dark fermentation process is characterized by low hydrogen yield, which consequently affects methane production. This phenomenon has been attributed to factors such as substrate and product inhibitions, environmental and operating parameters, or the tendency of the fermentation process to result in biomass production. The challenge with most of the hydrogen production pathways during dark fermentation is the problem of underutilization of substrate, with only one-third of the substrate having potential to be converted into hydrogen while the remaining two-thirds form organic acids and reduced compounds [6]. Moreover, some fermentative feedstocks often contain inhibitory compounds that tend to inhibit the feedstock degradability by anaerobic microorganisms.

Fruit waste has been widely utilized as feedstock during anaerobic fermentation due to its degradability and availability from the huge turnout of the wastes from human consumption and processing. However, the yields from the fruit fermentation processes are often low, which have been attributed, among other factors, to the flavor compounds inherently present in the fruits. Flavors are complex mixture of various organic compounds, including aldehydes, terpenoids, alcohols, ketones, lactones, and esters, with antimicrobial activity against a wide range of bacteria, yeasts, and molds [7,8,9,10,11,12,13]. The antimicrobial natures of fruit flavors are evident in their various applications including as food preservatives [14,15] and alternative medicines [11,16,17,18,19,20,21]. Previous research activities on the effect of fruit flavor have also confirmed the toxicity of fruit flavor compounds [22,23,24,25,26,27,28]. The toxicity of flavor compounds against bacteria probably comes from the hydrophobic quality of flavor compounds, which allows them to penetrate and bind with phospholipids of the bacterial cell membrane as well as other cell organelles, thereby making them water permeable [29,30,31,32,33,34]. The cell integrity is lost if the concentration of the accumulated flavor compound exceeds a tolerable limit. Although the adaptive potential of bacteria against flavor compounds has been reported [35,36,37], the hold-up time of the bacteria depends on the concentration of the flavor compounds and the exposure period of the bacteria to the flavor compounds, as the bacterial resistance cannot by itself be sustained for a long period of time [38]. Considering the hydrophobic nature of flavor compounds, a hydrophilic barrier can be created around the bacterial cells during the fermentation process to prevent direct bacterial contact with the flavor compounds as well as to reduce bacteria exposure time to the flavor compounds. The technique of employing a hydrophilic polyvinylidene fluoride (PVDF) barrier around an anaerobic microorganism in a medium containing flavor compounds, in order to reduce the antimicrobial effects of the flavor compounds during the fermentation process, formed the basis of this study. A PVDF membrane is a semi-crystalline polymeric membrane consisting of both crystalline and amorphous phases with the crystalline part responsible for its excellent thermal stability while the amorphous part is responsible for the flexibility of the membrane [39]. The membrane is chemically stable to a wide range of chemical compounds including inorganic acids, oxidants, halogens, aromatic, aliphatic, and chlorinated solvents. However, the intrinsic hydrophobic nature of PVDF makes it prone to organic fouling and low wettability, with high resistance to water flow. Consequently, several membrane modification techniques including blending, surface coating, irradiation grafting, and plasma modification are used to incorporate hydrophilicity into hydrophobic PVDF membranes to enhance their performance [40].

In several studies involving fermentation processes, cell encapsulation has been proved to be an effective technique for cell stability, high biomass concentration, and enhanced fermentative hydrogen and methane production [41,42]. Cell encapsulation is vital for cell survival and increased tolerance to toxic medium such as industrial wastewaters, which contain toxic compounds including phenols, benzenes, and halogenated aliphatics, among others [43]. Application of membrane in cell encapsulation has the potential of enhancing the total energy value of the fermentation process, which is among the main objectives of producing hydrogen and methane from the process [44,45]. Meanwhile, there have been no previous reports on the effects of using hydrophilic PVDF membranes for cell encapsulation on fermentative hydrogen from media containing hexanal, myrcene, and octanol flavors, although there are some reports on the protective effects of membrane encapsulation on fermentative methane production from limonene-contained media [22,23]. The objective of this study was therefore to investigate the potential of using membrane-encapsulated cells to improve hydrogen and methane productions from media containing hexanal, myrcene, and octanol during batch and semi-continuous fermentation processes. Enclosing fermentative microorganisms inside a hydrophilic membrane during the fermentation process could reduce the bacterial exposure to the antimicrobial effects of fruit flavors, minimize the penetration of the fruit flavors, and thereby improve the yields of hydrogen and methane productions. Moreover, information from further studies on the direct correlation between the concentration of flavor compounds and their corresponding antimicrobial effects could be applied in the health sector to combat the menace of malaria and dengue fever in tropical regions of the world. For example, Shan *et al.* (2007) reported that the antibacterial activity of extracts from dietary spices and medicinal herbs was closely associated with their phenolic constituents [46]. Also, the findings from the study conducted by Caccioni *et al.* (1998) showed a positive correlation between pathogen fungi inhibition and the content of the citrus fruit essential oil [47].

## 2. Results and Discussion

Effective fermentative methane production from fruit wastes during anaerobic digestion has been observed to be limited by the inherent fruit flavors, which act as the fruit’s defense mechanism against microbial invasion [22]. Since fermentative hydrogen is a precursor for methane production, it is likely that hydrogen production could also be limited by fruit flavors. Hexanal, myrcene, and octanol are fruit flavors that are naturally produced in fruits such as apple, grape, mango, orange, strawberry, and plum, which are essential parts of the human diet. Consequently, large quantities of the slowly digestible fruit wastes are generated from their production, processing, and consumption, thereby constituting environmental pollution and human health hazards. It is therefore necessary to devise a technique for improving degradation of the fruit wastes and thereby increase the hydrogen and methane production potential of the fruit wastes.

### 2.1. Effects of Fruit Flavors on Methane Production during the Batch Fermentation Process

**Figure 1:**
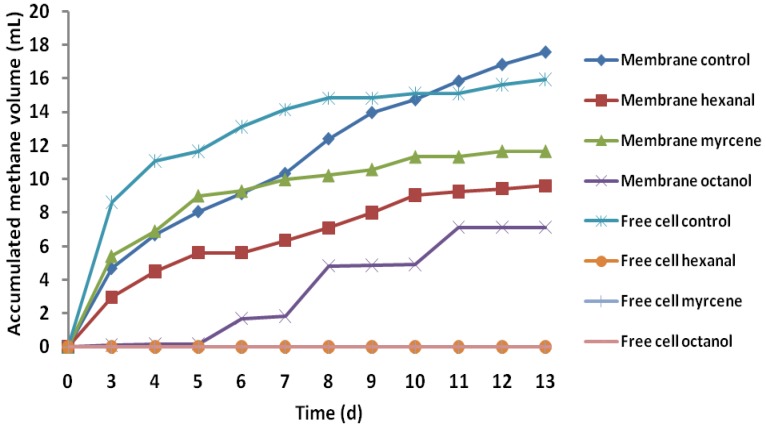
Batch fermentation process for accumulated methane production from substrate with fruit flavors in comparison with the control experiment (Table S1).

The batch fermentation process for methane production of encapsulated and free cells from a medium containing 0.5% w/v (5 g/L) concentration of fruit flavors including hexanal, myrcene, and octanol was carried out at 55 °C for 11 days, with manual mixing of the reactors twice a day. The results indicated no methane production from free cells directly in contact with all the fruit flavors at a concentration of 0.5% w/v (Figure 1). On the contrary, cumulative methane yields of 182 ± 15, 111 ± 81, and 150 ± 24 mL/g COD were obtained from the encapsulated bacteria immersed in medium with myrcene, octanol, and hexanal, respectively. The lowest methane yield was from octanol, indicating that the inhibitory effect of octanol seemed to be stronger than that of hexanal and myrcene. Although this could be related to the solubility, size, and chemical structure of the flavor compounds, which influence the flavor’s permeability, the mechanisms of inhibition during the fermentation process are sometimes difficult to understand, partly due to the various adaptive abilities of fermentative microorganisms.

Comparison of the accumulative methane production from encapsulated cells without flavor compounds (membrane control) and encapsulated cells with flavor compounds (membrane hexanal, membrane myrcene, and membrane octanol) indicates that the membrane’s protective effect against the flavor compounds could be given as approximately 60%. This implies that the membrane could protect more than half of the methane production from being affected by the inhibitory effect of the flavor compounds. The results, suggest, therefore, that encapsulated cells can withstand higher concentrations of fruit flavor than free cells, and it is therefore possible to have improved biomethane production from a fruit-flavored medium using membrane-cell encapsulation.

### 2.2. Effects of Fruit Flavors on Hydrogen Production during the Semi-Continuous Fermentation Process

**Figure 2:**
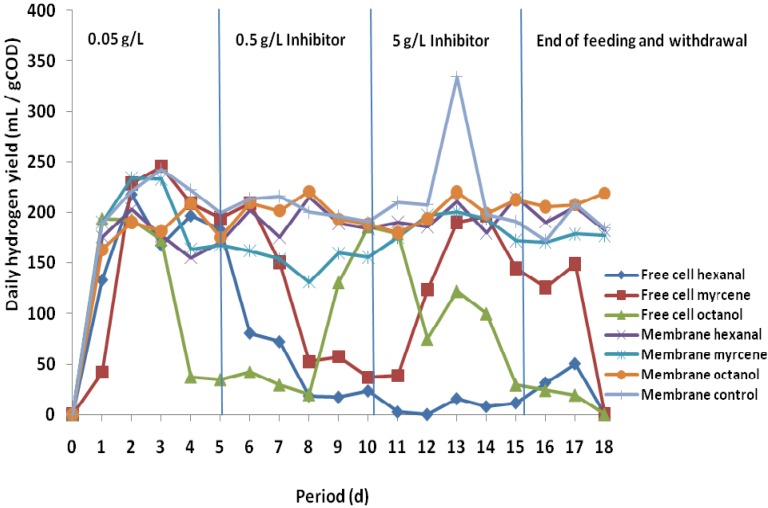
Semi-continuous fermentation process for daily hydrogen yield from substrate with fruit flavors in comparison with the control experiment (Table S2).

**Figure 3:**
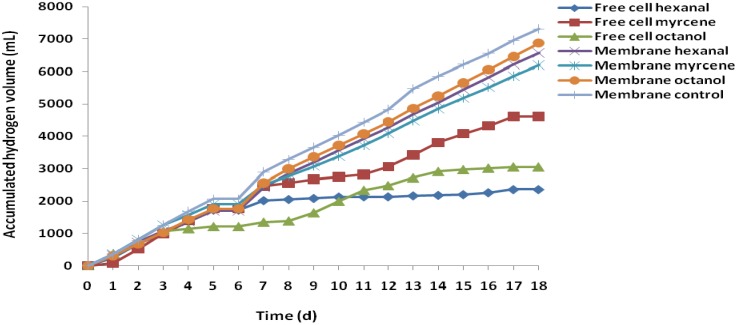
Semi-continuous fermentation process for cumulative hydrogen volume from substrate with flavors in comparison with the control experiment (Table S3).

The inhibitory effects of three flavor compounds (hexanal, myrcene, and octanol) on hydrogen production potentials of fermentative microorganisms were investigated during the semi-continuous fermentation operated for 18 days at 55 °C. The concentrations of the fruit flavor compounds were increased at intervals of five days, starting with 0.05 g/L through 0.5 and finally 5 g/L. After the 15th day of the fermentation process, the feed influent and effluent withdrawal from the fermentation system were ceased for three days in order to observe how the system adjusts to the inhibitory effects of the flavors in the fermentation medium. The average daily yields (Figure 2) and accumulated volumes of hydrogen (Figure 3) obtained from the fermentation process clearly showed the protective effects of employing encapsulated cells during anaerobic fermentation process. The average hydrogen production from the encapsulated cells was higher than the production from the free cells. Meanwhile, none of the flavor compounds used during the semi-continuous fermentation process could be said to have the greatest inhibitory effect, as the degree of the inhibitory effects varied among the fruit flavors. For example, octanol was found to have the greatest inhibitory effect in the batch fermentation process, while both hexanal and myrcene were observed to have greater inhibitory effects than octanol in the semi-continuous process. When the free cells were exposed to the flavor compounds, hexanal showed the greatest inhibitory effect, as indicated by the low average daily hydrogen yield of 68mL/g COD, while among the encapsulated cells, myrcene showed the lowest average daily hydrogen yield of 179 mL/g COD. The variation could be due to the complexity of antimicrobial mechanisms of flavor compounds coupled with the adaptive potential of the fermentative microorganisms.

At flavor concentration of 0.05 g/L, the inhibitory effects of the flavor compounds were not significant, as the average hydrogen yields from the encapsulated and free cells were almost the same (Table 1). However, the inhibitory effects of the flavor compounds, especially among the free cells, were considerably significant when the concentration was increased to 0.5 g/L. The percentage reduction in average daily hydrogen yields from hexanal, myrcene, and octanol were 77, 45, and 35%, respectively (Table 2). The increase in the flavor concentration did not have much effect on encapsulated cells except when myrcene was used as the flavor, which resulted in the reduction of the average daily hydrogen yield by 23%. When the flavor concentration was increased from 0.5 to 5 g/L, there was a corresponding increase in the average daily hydrogen yields (Table 2) from both encapsulated and free cells, indicating an improvement in the activities of the cells. The reason might be the adaptive ability of the anaerobic microorganisms to the inhibitory medium as well as the potential of the microorganisms to degrade some of the flavor compounds. Meanwhile, when the supply of nutrient and withdrawal of effluent stopped, the average daily hydrogen production from the free cells dropped significantly, except for free cells in hexanal medium, which experienced yield increase. However, the average daily yields from the encapsulated cells did not experience much change three days after ending the feed supply and withdrawing the effluent.

**Table 1:** Average hydrogen yields at the three flavor concentrations.

**Fermentation Media**		**Average Hydrogen Yield (mL/g COD)**			
		0.05 g/L Flavor Concentration	0.5 g/L Flavor Concentration	5 g/L Flavor Concentration	No Feeding and Withdrawal
(A)	*Free cells*	-	-	-	-
	Hexanal	179.6	42.3	7.55	27.23
	Myrcene	183.9	101.2	138.7	91.53
	Octanol	126.2	81.5	100.9	14.4
(B)	*Membrane*	-	-	-	-
	Hexanal	176.5	193.2	196.4	192.7
	Myrcene	197.9	152.5	187.9	175.6
	Octanol	183.8	202.7	200.9	210.8

**Table 2:** Effects of change in flavor concentration on average hydrogen yield.

**Fermentation Media**		**Change in Average Hydrogen Yield (%)**		
		Increase of Flavor Concentration from 0.05 to 0.5 g/L	Increase of Flavor Concentration from 0.5 to 5 g/L	Reduction of Flavor Concentration from 5 to 0 g/L (no Feeding and Withdrawal)
(A)	*Free cells*	-	-	-
	Hexanal	(−) 77	(−) 82	(+) 72
	Myrcene	(−) 45	(+) 27	(−) 34
	Octanol	(−) 35	(+) 19	(−) 85
(B)	*Membrane*	-	-	-
	Hexanal	(+) 9	(+) 2	(−) 2
	Myrcene	(−) 23	(+) 19	(−) 7
	Octanol	(+) 9	(−) 1	(+) 5

Notes: − reduction; + increase.

Throughout the experiment, it might be worthwhile to state that the increase in the concentration of flavor compound in the fermentation medium did not significantly affect the average daily hydrogen yield from the encapsulated cells, as the hydrogen production was nearly constant. It was also observed that, although free cells of hydrogen-producing bacteria were able to produce reasonable amounts of hydrogen regardless of the flavor inhibitors, the amount of hydrogen produced was less, compared to encapsulated cells. Based on the results, it could therefore be concluded that fermentative hydrogen and methane production from an inhibitory fruit-flavored medium could be improved using the technique of membrane-cell encapsulation.

### 2.3. Digestate pH Values during the Semi-Continuous Fermentation Process

**Figure 4:**
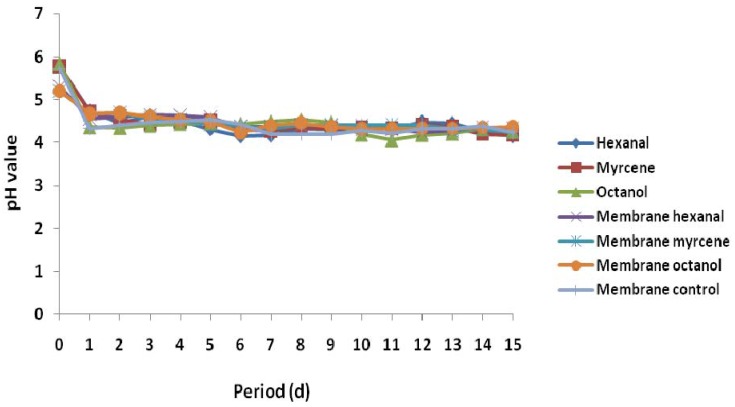
Daily digestate pH values during the semi-continuous fermentation process (Table S4).

The pH plays an important role during fermentative hydrogen production as it affects the metabolic pathways in hydrogen production as well as limits hydrogen consumption by hydrogenotrophic methanogens [48,49,50,51]. Hydrogen and methane production during the fermentation process requires different pH values of 5.5–6.5 and 6.5–8.2, respectively. In this study, batch fermentation was used for methane production with the pH range of 6.8–7.2, while semi-continuous fermentation was used for hydrogen production with a range of initial pH range values of 5.2 to 5.9 [52]. During the semi-continuous fermentation, gradual reduction in the pH values of the fermenting media below 5.0 was observed at the beginning of the experiment, which could be attributed to the production of organic acids associated with the hydrogen formation during the fermentation process [53]. The pH profile (Figure 4) indicated that the pH values for all the reactors did not vary significantly but were nearly constant throughout the experiment, with an average value of 4.40 ± 0.04 (Table S4). This could possibly imply that the daily effluent withdrawal from the reactor system could have prevented the accumulation of organic acids, which could have led to a drastic reduction in the pH value of the fermentation media. Moreover, it could also be due to the adaptive potential of fermentative microorganisms to the inhibitory fermentative media.

### 2.4. Implication of Membrane Applications for Cell Encapsulation

Encapsulation techniques could have some limitations including the inefficient diffusion of nutrients to the microorganisms in the membrane as well as membrane fouling. It is often necessary to determine the water permeability of the membranes to be employed during the fermentation process. The permeability results can also be useful in the determination of the loss in membrane efficiency after the fermentation process. In this study, an average value of 0.048 mL/min of pure water permeability was obtained for the PVDF membrane. This indicates that in a time period of one minute, the membrane could allow an approximate value of 0.048 mL of distilled water to pass through it. Membrane permeability is influenced by various factors including the membrane materials (pore size, hydrophobicity/hydrophilicity, free volume, and filler particles) and the solubility of the permeates [54,55,56].

The resistance to fluid flow through the membrane during the filtration process is often due to membrane fouling, which is a term used to describe the loss of membrane throughput. Generally, fouling occurs when particulate, colloidal, or soluble materials are deposited inside the membrane pores or surface. Membrane fouling is a major barrier to membrane application in fermentation processes, as it is associated with flux or permeate flow reduction, low permeate quality, and increased operational costs due to increased energy consumption. Membrane fouling is influenced by factors such as sludge characteristics, operational parameters, and membrane qualities. Although membrane fouling cannot be entirely avoided during the membrane filtration process, the frequency of its occurrence could be reduced through physical cleaning such as relaxation and backwashing or chemical cleaning. Chemical cleaning of the membrane is more effective in removing membrane fouling than physical cleaning, but frequent use of chemical cleaning can damage the membrane and shorten the lifetime of the membrane. Previously, membrane cost was part of the barrier to the application of membrane technology, but extensive research on membrane improvement has resulted in cheaper and more affordable membranes in recent times. However, the operating costs associated with membrane fouling abatement are still a major barrier to the application of membrane technology.

## 3. Experimental Section

### 3.1. Materials

#### 3.1.1. Anaerobic Sludge

The anaerobic sludge used for the digestion during the study was an effluent sludge obtained from an active 3000 m^3^ municipal solid waste thermophilic (55 °C) digester at Borås Energy and Environment AB (Borås, Sweden). Prior to the start of the experiment, the sludge was incubated at 55 °C for three days before it was employed as an inoculum during the study to ensure that the biogas production from the active bacteria in the sludge ha stopped or reduced before the commencement of the experiment. After the incubation, the sludge was thoroughly mixed and filtered with a screen of 1 mm pore size to remove particles bigger than the pore size of the screen. For encapsulation purposes, the sludge was centrifuged at 14,000× g for 10 min to separate the solid inoculum from the supernatant [22].

#### 3.1.2. Membrane Encapsulation Procedure

The synthetic encapsulating sachets for holding the bacteria were made of flat sheet hydrophilic polyvinylidene fluoride (PVDF) membranes (Durapore^®^, Thermo Fisher Scientific Inc., Stockholm, Sweden) with pore size, thickness, and diameter of 0.1 μm, 125 μm, and 90 mm, respectively. The encapsulating sachets were prepared as described in a previous report [22]. Each membrane was cut and folded into rectangular dimensions with a width and length of 3 and 6 cm, respectively. The membranes were heat-sealed (HPL 450 AS, Hawo, Germany) with heating and cooling times of 5.5 s while leaving one side left open for cell insertion, after which the opening was sealed to form a membrane capsule. The sealing and cooling times for the membranes were 5.0 and 5.5 s, respectively. The fermentation process was carried out immediately after the membrane encapsulation procedure was completed.

#### 3.1.3. Nutrient Medium and Flavor Compounds

The nutrient medium used during the fermentation process was a synthetic medium consisting of 20 g/L glucose (supplied by Merck, Millipore, Darmstadt, Germany), 20 g/L yeast extract (supplied by Merck, Millipore, Darmstadt, Germany), and 20 g/L nutrient broth (supplied by Sigma-Aldrich, Stockholm, Sweden). The nutrient broth contained D-(+)-glucose (1 g/L), peptone (15 g/L), sodium chloride (6 g/L), and yeast extract (3 g/L). The medium was sterilized by filtration through a 0.2-μm membrane before it was used for the fermentation process. The flavor compounds (supplied by Sigma-Aldrich, Stockholm, Sweden), consisting of hexanal, myrcene, and octanol, were used as inhibitors during the fermentation process.

### 3.2. Experimental Setup and Procedures

The experiment was separated into two parts. The first part was batch fermentation for methane production while the second part was the semi-continuous fermentation process for hydrogen production. Both fermentation processes were carried out under thermophilic conditions (55 °C), and the same flavor compounds, including hexanal, myrcene, and octanol, were used. The seed inoculum was incubated at 55 °C for three days before it was employed for both batch and semi-continuous fermentation processes [57].

#### 3.2.1. Batch Fermentation Process for Methane Production

The reactors used for the batch fermentation of methane were 118 mL serum glass bottles with an active volume of 53.5 mL and a headspace of 65.5 mL. Each reactor was filled with 1.0 mL of filtered nutrient medium containing 20 g/L each of nutrient broth, yeast extract, and D-(+)-glucose. Three fruit flavor compounds, hexanal, myrcene, and octanol, were used, each having a 0.5% w/v concentration prepared by dissolving 5 g of the inhibitor in 1 liter of distilled water. Fifty milliliters of the anaerobic sludge were measured and centrifuged, from which a 3-g pellet was used for the encapsulation. For each flavor investigated, the batch fermentation reactors were grouped into two categories, encapsulated or free cells. For encapsulated cells with an inhibitor, the reactor bottle contained 3 g of the inoculum pellet encapsulated in the membrane, 47 mL of distilled water, 2.5 mL of the flavor compound (0.5% w/v), and 1 mL of nutrient medium. Regarding the free cells with an inhibitor, the reactor bottle contained 50 mL of uncentrifuged inoculum, 2.5 mL of the flavor compound, and 1 mL of nutrient medium. Besides the two groups of reactor bottles, other groups included membrane and free cells controls, both of which differed from the first two groups by the replacement of the fruit flavor with 2.5 mL of distilled water. Blank reactors containing 50 mL of non-centrifuged inoculum and 3.5 mL of distilled water were also prepared. After filling the serum glass bottles with an appropriate medium of pH between 6.8 and 7.2, they were closed with rubber seals and plastic caps. The bottle headspace was flushed with 80% nitrogen and 20% carbon dioxide to create an anaerobic environment [58]. The batch fermentation process for methane production of encapsulated and free cells from a medium containing 0.5% w/v (5 g/L) concentration of fruit flavors including hexanal, myrcene, and octanol was carried out at 55 °C for 11 days, with manual mixing of the reactors twice a day to enhance the fermentation activities.

#### 3.2.2. Semi-Continuous Fermentation Process for Hydrogen Production

The semi-continuous experiments were carried out using parallel 500 mL bioreactors and a data acquisition system (AMPTS, Bioprocess Control Sweden AB, Lund, Sweden). Prior to the start of the semi-continuous experiment, the sludge for the fermentative hydrogen production was heat-pretreated at 100 °C for 15 min and the initial pH adjusted to values between 5.2 and 5.9, as hydrogen production has been observed to be enhanced at the pH range [59]. A pellet of the inoculum sludge with an average weight of 32 g (equivalent to 5.6 g VSS/L) obtained from the centrifuged sludge was used separately for each reactor with free and encapsulated cells. Regarding the reactors with free cells, the inoculum pellet (5.6 g VSS/L) was added into each 500-mL glass reactor bottle (liquid volume of 450 mL) containing 300 mL of filtered nutrient medium and 97 mL of distilled water. The nutrient medium was composed of 20 g/L each of nutrient broth, yeast extract, and D-glucose. The resulting mixture was thoroughly mixed so that the inoculum pellet could dissolve completely to form homogeneous mixture. The flavor compounds (myrcene, octanol, and hexanal) were prepared in three different concentrations, 0.05, 0.5, and 5 g/L, after which 21 mL of the prepared flavor solutions were added into each reactor. For encapsulated cell-reactors, the inoculum pellet (32 g) was divided into eight equal portions (4 g each), which were inserted into eight membrane sachets. Each reactor bottle contained eight membrane sachets, with each sachet enclosing 4 g of inoculum pellet. The whole experiment was started with the addition of lowest flavor concentration (0.05 g/L) while the gradual increase in concentration was done at an interval of five days. With the constant active volume of 450 mL and daily flow of 50 mL/d, the hydraulic retention time (HRT) throughout the experiment was nine days. Throughout the experiment, the reactor bottles were shaken twice a day to ensure adequate contact among the nutrients, anaerobic cells, and flavor compounds. The pH of the effluent withdrawn on each day of the experiment was measured in order to gain insight into the state of the fermentation process during the experiment.

### 3.3. Analytical Method

The volumes of biogas and hydrogen generated during the anaerobic fermentation processes were measured using a data acquisition system (AMPTS, Bioprocess Control Sweden AB, Lund, Sweden). The individual gas compositions were determined by using a 0.25-mL syringe (VICI, precious sampling Inc., Baton Rounge, LA, USA) for the gas sampling while the gas quantification was done using a gas chromatograph (Perkin-Elmer, Shelton, CT, USA). The gas chromatograph was equipped with a packed column (Perkin-Elmer, 6′ × 1.8″ OD, 80/100, Mesh, Shelton, CT, USA) and a thermal conductivity detector (Perkin-Elmer, Shelton, CT, USA) with an inject temperature of 150 °C. Nitrogen, at a flow rate of 20 mL/min at 60 °C, was used as the carrier gas.

### 3.4. Membrane Performance Measurement

The ability of a membrane to regulate the permeation of various molecules through it is an important feature that is employed in separation processes. The permeation process can either follow the solution–diffusion model, where the permeants dissolve and diffuse through the membrane, or the pore flow model, where the permeants pass through the membrane pores. The driving forces producing movement of permeants, which could be concentration, pressure, temperature, or electromotive force, are connected in such a way that overall driving force is the chemical potential [60]. Membrane permeability determines the rates of movement of nutrients and inhibitors into the cells of the fermentative microorganisms as well as the discharge of the cell metabolism products. In this study, distilled water was used to determine the pure water permeability (PWP) parameter of the hydrophilic PVDF membranes used during the experimental work in this study. The time required for a definite quantity of distilled water to pass through the membranes was observed and recorded. The water flow rate through the membrane was calculated by dividing the volume of permeated water by the time required for the permeation. Since the experiment was carried out at room temperature (22 °C), a temperature correction of 0.794 was used to adjust the values obtained from the permeability test.

### 3.5. Estimation of Chemical Oxygen Demand of the Substrate

The substrate used for the fermentation process was a synthetic medium consisting of 20 g/L glucose (supplied by Merck, Millipore, Darmstadt, Germany), 20 g/L yeast extract (supplied by Merck, Millipore, Darmstadt, Germany) and 20 g/L nutrient broth (supplied by Sigma-Aldrich, Stockholm, Sweden). The concentration of the substrate carbon source was, therefore, assumed to be 60 g/L., such that the amount of glucose in 1000 mL of the nutrient medium was equivalent to 60g. By applying Equation (1), 1 g/mol glucose (C_6_H_12_O_6_) is equivalent to 1.07g COD. For batch fermentation, 1 mL of the nutrient medium was used, which was estimated to contain 0.0642 g COD, while in the semi-continuous fermentation process, 30mL of the nutrient medium containing 1.926 g COD was used daily.

(1) $$C_{6}H_{12}O_{6}+6O_{2}\to 6CO_{2}+6H_{2}O.$$

## 4. Conclusions

The major barriers associated with the widespread applications of fermentative hydrogen and methane as fuels include, among others, the low yields of the gas production. The low yields have been partly attributed to substrate inhibition. This study, therefore, investigated the inhibitory effects of some flavor compounds in fruits, which is one of the important factors contributing to low hydrogen and methane yields during the fermentation process of fruit wastes. The potential of employing membrane technology to improve the yields of hydrogen and methane from such a process was explored. The results suggest that the membrane-based techniques could actually improve hydrogen and methane production from fermentation media with substrate inhibition. Compared with the free cells, membrane-encapsulated cells produced methane faster and were able to survive the effects of the inhibitory flavor medium. Higher gas production was also observed from encapsulated cells, when compared to free cells, in the inhibitory fruit flavor. However, it could be observed from the results obtained that the membrane could not completely protect the fermentative organism against the inhibitory effects of flavor compounds. Therefore, further membrane improvement is necessary to effectively protect the microorganism from the inhibitory fruit flavor medium. Large-scale application of the membrane-encapsulated technique for effective fermentative hydrogen and methane productions is feasible since cell microencapsulation technology has been successfully applied on a large scale in other fields including food industry and medicine. For example, in the food industry, encapsulation of live probiotic bacterial cells was used to increase the bacterial viability during processing of dairy products [61].

## Supplementary Files

Supplementary File 1
