# Supplementary material for: Enhanced Fermentative Hydrogen and Methane Production from an Inhibitory Fruit-Flavored Medium with Membrane-Encapsulated Cells
Source: Membranes (Basel). 2015 Oct 16;5(4):616–31. doi: 10.3390/membranes5040616 (PMC4704002; doi:10.3390/membranes5040616)
Supplement: Supplementary File 1 [file membranes-05-00616-s001.pdf]

Supplementary Materials

Table S1. Cumulative methane volume (mL) for batch process.

**Fermentation media**	**Cumulative methane volume (mL)**										
**Days**	**3**	**4**	**5**	**6**	**7**	**8**	**9**	**10**	**11**	**12**	**13**
Membrane control (A1)	5.124	2.255	2.155	1.167	2.766	3.454	2.765	1.754	1.476	1.682	1.434
Membrane control (A2)	4.806	1.834	0.875	0.390	2.154	2.455	1.922	0.553	1.576	0.644	0.308
Membrane control (A3)	4.133	1.929	1.105	1.673	0.024	0.291	-0.525	-0.836	0.249	0.662	0.469
AVERAGE	4.688	2.006	1.378	1.077	1.648	2.067	1.388	0.490	1.100	0.996	0.737
STD	0.506	0.221	0.682	0.646	1.439	1.617	1.709	1.296	0.739	0.594	0.609
Membrane hexanal (C1)	3.750	1.717	1.610	0.322	1.934	1.286	1.742	1.089	-1.198	0.225	-5.065
Membrane hexanal (C2)	2.651	1.688	0.712	-0.606	-0.513	-0.566	1.538	0.936	-0.423	1.235	-0.828
Membrane hexanal (C3)	2.484	1.493	1.520	-0.268	1.553	2.732	-0.877	1.048	0.015	-1.414	7.277
AVERAGE	2.961	1.633	1.281	-0.184	0.991	1.150	0.801	1.024	-0.536	0.015	0.461
STD	0.688	0.122	0.495	0.470	1.317	1.653	1.457	0.080	0.614	1.337	6.272
Membrane myrcene (E1)	5.300	2.164	2.568	-0.682	1.994	0.770	-0.105	1.040	-0.059	0.645	-0.843
Membrane myrcene (E2)	5.089	1.616	2.149	1.477	1.619	-0.346	1.037	0.920	-0.848	-0.724	-1.064
Membrane myrcene (E3)	5.861	1.702	2.100	2.359	1.865	0.528	-0.785	0.350	-1.607	0.753	-1.839
AVERAGE	5.417	1.827	2.272	1.051	1.826	0.318	0.049	0.770	-0.838	0.225	-1.249
STD	0.399	0.294	0.257	1.565	0.191	0.587	0.921	0.368	0.774	0.823	0.523
Membrane Octanol (F1)	0.165	0.096	-0.429	0.123	0.221	-0.104	0.092	0.057	4.469	0.000	0.000
Membrane Octanol (F2)	0.076	0.018	-0.034	2.921	0.039	6.002	-0.062	0.001	0.616	0.000	0.000
AVERAGE	0.120	0.057	-0.232	1.522	0.130	2.949	0.015	0.029	2.543	0.000	0.000
STD	0.063	0.056	0.280	1.978	0.129	4.318	0.109	0.039	2.724	0.000	0.000
Free cell Control (H1)	11.890	3.194	1.370	2.287	-1.136	1.597	-1.225	0.690	-1.225	0.658	-0.636
Free cell Control (H2)	12.827	2.304	0.529	2.060	1.789	0.448	-1.427	-4.443	-1.427	1.502	-0.909
Free cell Control (H3)	11.152	2.893	0.855	-0.115	1.271	-0.510	-0.942	0.056	-0.942	1.430	1.996
AVERAGE	11.956	2.797	0.918	1.411	0.641	0.512	-1.198	-1.232	-1.198	1.197	0.151
STD	0.839	0.452	0.424	1.326	1.561	1.055	0.243	2.798	0.243	0.468	1.604
Free cell Hexanal (I1)	-12.516	-3.142	-3.519	-2.699	-1.892	-2.941	-2.311	-3.740	-2.311	-2.114	0.293

Table S1. *Cont.*

Fermentation media	Cumulative methane volume (mL)										
**Days**	**3**	**Days**	**3**	**Days**	**3**	**Days**	**3**	**Days**	**3**	**Days**	**3**
Free cell Hexanal (I2)	-8.543	-2.873	-2.309	-1.563	5.137	-2.741	-3.092	-4.952	-3.092	-1.461	-1.641
Free cell Hexanal (I3)	-11.636	-2.898	-3.865	-2.993	-1.783	-3.367	-2.181	-3.848	-2.181	-2.611	-1.199
AVERAGE	-10.898	-2.971	-3.231	-2.418	0.487	-3.016	-2.528	-4.180	-2.528	-2.062	-0.849
STD	2.09	0.15	0.82	0.76	4.03	0.32	0.49	0.67	0.49	0.58	1.01
Free cell myrcene (J1)	-1.13	-1.29	-3.31	-1.43	-1.09	-2.69	-3.38	-4.09	-3.38	-1.82	-1.06
Free cell myrcene (J2)	-13.01	-2.75	-2.89	-1.31	-1.98	-2.83	-1.79	-3.44	-1.79	-1.87	-0.61
Free cell myrcene (J3)	-1.34	-0.97	-4.21	-1.93	-0.39	-2.82	-2.94	-4.03	-2.94	-2.33	0.08
AVERAGE	-5.16	-1.67	-3.47	-1.56	-1.15	-2.78	-2.71	-3.85	-2.71	-2.01	-0.53
STD	6.79	0.95	0.68	0.33	0.80	0.08	0.82	0.36	0.82	0.28	0.58
Free cell octanol (K1)	1.16	-2.72	-3.77	-1.78	-2.16	-3.43	-2.06	-3.28	-2.06	-1.28	0.58
Free cell octanol (K2)	4.59	1.37	-3.99	-3.50	-1.20	-2.87	-2.40	-2.99	-2.40	-2.54	-0.04
Free cell octanol (K3)	4.22	-4.67	-3.98	-1.47	0.08	-3.05	-1.84	-5.50	-1.84	-6.06	-1.25
AVERAGE	3.32	-2.01	-3.91	-2.25	-1.09	-3.12	-2.10	-3.92	-2.10	-3.30	-0.24
STD	1.88	3.09	0.12	1.09	1.12	0.29	0.28	1.37	0.28	2.48	0.93

Table S2. Daily hydrogen yield from the continuous process.

**Fermentation media**		**0.05 g/L flavour compound**						**0.5 g/L flavour compound**			
**Days**	**0**	**1**	**2**	**3**	**4**	**5**	**6**	**7**	**8**	**9**	**10**
Free cell hexanal	0	133.071	217.8106	167.6776	196.6744	182.9788	80.48738	72.01811	18.5106	17.18856	23.41865
Free cell myrcene	0	41.75176	228.7156	245.5282	209.0187	194.4635	208.9919	150.6967	52.33569	57.23433	36.91765
Free cell octanol	0	193.4833	192.4834	172.7883	37.30913	34.71106	41.95425	29.43021	19.8704	130.5985	185.5679
Membrane hexanal	0	175.8661	203.3634	177.353	155.2915	170.7257	201.9938	175.7138	215.2445	188.8841	184.2344
Membrane myrcene	0	190.3026	234.1959	233.191	163.8689	167.948	162.0394	153.7609	131.2461	159.7315	155.7994
Membrane octanol	0	163.1317	190.2278	181.3317	209.122	175.2816	209.3723	201.6011	220.681	193.2967	188.5383
Membrane control	0	189.5333	221.2776	242.5365	221.9434	199.6217	213.372	216.0065	200.1613	196.073	190.5891

**Fermentation media**	**5 g/L flavour compound**				**No feeding and withdrawal**			
**Days**	**11**	**12**	**13**	**14**	**15**	**16**	**17**	**18**
Free cell hexanal	2.565861	0.109095	15.66905	7.90815	11.47884	31.33813	50.36318	0
Free cell myrcene	38.49031	123.4181	190.2912	196.4132	144.7689	125.6641	148.9368	0
Free cell octanol	178.9586	74.56774	121.8587	99.74473	29.36923	23.88918	19.34015	178.9586
Membrane hexanal	190.4631	185.7963	210.7285	180.0042	214.8009	190.5264	205.3442	182.344
Membrane myrcene	175.6045	197.4283	200.6161	193.5896	172.2102	170.7286	178.6694	177.2652
Membrane octanol	179.9169	193.6439	220.2881	198.5666	212.2672	205.6174	207.7396	219.0001
Membrane control	209.9718	207.3753	333.7825	198.0239	190.9691	172.2271	208.5333	184.397

Table S3. Cumulative hydrogen volume (mL) from the continuous process.

**Fermentation media**	**0.05 g/L flavour compound**						**0.5 g/L flavour compound**				
**Days**	**0**	**1**	**2**	**3**	**4**	**5**	**6**	**7**	**8**	**9**	**10**
Free cell hexanal	0	256.2947	675.7979	998.7449	1377.54	1729.957	1729.957	2023.683	2059.334	2092.439	2137.543
Free cell myrcene	0	80.4139	520.9201	993.8075	1396.378	1770.914	1770.914	2463.674	2564.473	2674.706	2745.81
Free cell octanol	0	372.6487	743.3719	1076.162	1148.019	1214.873	1214.873	1352.359	1390.63	1642.163	1999.566
Membrane hexanal	0	338.7181	730.396	1071.978	1371.069	1699.887	1699.887	2427.352	2841.913	3205.704	3560.539
Membrane myrcene	0	366.5229	817.5843	1266.71	1582.322	1905.789	1905.789	2514.021	2766.801	3074.443	3374.513
Membrane octanol	0	314.1916	680.5704	1029.815	1432.584	1770.177	1770.177	2561.712	2986.743	3359.032	3722.157
Membrane control	0	365.0412	791.2218	1258.347	1685.81	2070.281	2070.281	2897.264	3282.775	3660.412	4027.486

**Fermentation media**	**5g/L flavour compound**					**No feeding and withdrawal**		
**Days**	**11**	**12**	**13**	**14**	**15**	**16**	**17**	**18**
Free cell hexanal	2142.485	2142.695	2172.874	2188.105	2210.213	2270.571	2367.57	2367.57
Free cell myrcene	2819.942	3057.645	3424.146	3802.438	4081.263	4323.292	4610.144	4610.144
Free cell octanol	2344.241	2487.858	2722.558	2914.666	2971.231	3017.242	3054.491	3054.491
Membrane hexanal	3927.371	4285.215	4691.078	5037.766	5451.472	5818.426	6213.919	6565.113
Membrane myrcene	3712.727	4092.974	4479.361	4852.214	5183.891	5512.714	5856.832	6198.244
Membrane octanol	4068.677	4441.635	4865.91	5248.349	5657.176	6053.195	6453.302	6875.096
Membrane control	4431.892	4831.297	5474.162	5855.556	6223.362	6555.072	6956.707	7311.855

Table S4. pH measurement of effluent from the continuous hydrogen production.

**Fermentation media**	**pH values**															
**Days**	**0**	**1**	**2**	**3**	**4**	**5**	**6**	**7**	**8**	**9**	**10**	**11**	**12**	**13**	**14**	**15**
Free cell hexanal	5.77	4.74	4.45	4.63	4.52	4.31	4.14	4.16	4.32	4.31	4.3	4.33	4.48	4.45	4.25	4.14
Free cell myrcene	5.77	4.72	4.53	4.43	4.49	4.49	4.31	4.28	4.35	4.33	4.35	4.33	4.42	4.37	4.2	4.18
Free cell octanol	5.85	4.36	4.35	4.4	4.44	4.46	4.44	4.49	4.53	4.47	4.19	4.06	4.18	4.21	4.32	4.26
Membrane hexanal	5.31	4.56	4.61	4.64	4.63	4.58	4.36	4.36	4.41	4.39	4.32	4.32	4.23	4.28	4.35	4.33
Membrane myrcene	5.2	4.67	4.7	4.57	4.52	4.43	4.38	4.33	4.4	4.41	4.4	4.41	4.41	4.32	4.29	4.23
Membrane octanol	5.2	4.67	4.69	4.6	4.53	4.5	4.23	4.4	4.46	4.37	4.32	4.32	4.33	4.33	4.35	4.36
Membrane control	5.72	4.33	4.4	4.48	4.49	4.54	4.43	4.2	4.2	4.2	4.28	4.22	4.33	4.32	4.38	4.24

© 2015 by the authors; licensee MDPI, Basel, Switzerland. This article is an open access article distributed under the terms and conditions of the Creative Commons Attribution license (http://creativecommons.org/licenses/by/4.0/).
